# The impact of implementation of sex education in schools, and its applicability in Kazakhstan

**DOI:** 10.3389/fgwh.2026.1761178

**Published:** 2026-04-24

**Authors:** Saule Derbisbek, Aigul Abduldayeva, Abzal Mombekov, Zaituna Khamidullina, Gulnur Doszhanova

**Affiliations:** 1 Department of Research Institute of Preventive Medicine Named Academician E. Dalenov, Astana Medical University, Astana, Kazakhstan; 2 Department of Obstetrics and Gynecology, Astana Medical University, Astana, Kazakhstan; 3 Department of Obstetrics and Gynecology, Multidisciplinary City Hospital, Astana, Kazakhstan

**Keywords:** adolescents, comprehensive sexuality education, reproductive health, sexual health, STI prevention

## Abstract

**Background:**

In developing countries, including Kazakhstan, formal sex education remains limited due to sociocultural norms, insufficient resources, and the absence of standardized curricula.

**Objective:**

This systematic review evaluates the effectiveness of school-based comprehensive sex education (CSE) interventions on adolescents' knowledge, attitudes, and sexual behaviors, and assesses their applicability within the Kazakhstani context.

**Methods:**

A systematic literature search was conducted in PubMed, PMC, Cochrane, Scopus, Web of Science, and Google Scholar for studies published up to October 2025. Eligible studies included interventions delivering CSE to children or adolescents and reporting outcomes related to knowledge, attitudes, or sexual behaviors. Primary outcomes included sexual and reproductive health knowledge, sexual behaviors, and biological outcomes (STIs, adolescent pregnancies), secondary outcomes included attitudes toward consent, gender equity, and psychosocial measures.

**Results:**

The search did not yield any studies from Kazakhstan or neighboring countries pertaining CSE. However, twelve studies met the inclusion criteria, including eight cluster-randomized controlled trials, two quasi-experimental studies, one cross-sectional study and a longitudinal observational study conducted across multiple countries. Overall, the included studies consistently reported improvements in sexual health knowledge and more favorable attitudes toward safe sexual practices following CSE interventions. A number of studies also indicated positive behavioral trends, such as delayed sexual initiation, increased contraceptive use, and reductions in risky sexual behaviors (e.g., multiple partners and unprotected intercourse), although the magnitude of these effects varied across studies.

**Conclusions:**

School-based CSE interventions appear to improve adolescents' knowledge, attitudes, and sexual behaviors in diverse contexts. Factors such as sociocultural might hinder the implementation of CSE in Kazakhstan. However, findings are based on heterogeneous study designs and should be interpreted with caution. At the same time, a culturally sensitive and context specific pilot CSE programs integrated into Kazakhstan's school curriculum, alongside teacher training and supportive health services is warranted.

## Introduction

In developed countries, adolescents reportedly engaging in sexual activity at progressively earlier ages, due to a range of factors, including socioeconomic conditions, family and cultural expectations (1). In the USA, half of the two million sexually transmitted infections diagnosed annually, and more than 50% of unintended pregnancies reported among teenagers, with some estimates reaching up to 82% (2). However, measures such targeted awareness programs or educational interventions such as sex and reproductive education may help address these challenges. For instance, sex education in schools has been shown to produce measurable benefits for adolescent health, including delayed initiation of sexual activity, increased contraceptive use, and reductions in unintended pregnancies and sexually transmitted infections (STIs) (3, 4). Evidence also demonstrates that such programs improve sexual health knowledge, communication skills, and attitudes toward consent without increasing sexual activity among adolescents (5). Overall, the U.S. experience highlights that evidence-based comprehensive sex education (CSE) plays a critical role in promoting adolescent sexual and reproductive health (SRH) and advancing public health objectives (3, 5).

CSE is widely recognized as a critical component of adolescent health promotion, equipping young people with the knowledge and skills needed to make informed decisions about sexual behavior, relationships, and reproductive health. School-based CSE programs were shown to improve sexual health literacy, delay initiation of sexual activity, reduce risk-taking behaviors, and lower rates of unintended pregnancy and STIs (2, 6). Schools represent an accessible and influential setting for delivering structured educational interventions, making them central to national strategies aimed at safeguarding adolescent health.

To the contrary, sexuality education in developing countries is often limited by cultural norms, insufficient resources, and the absence of formal policies that support comprehensive reproductive health curricula in schools (7). Many programs rely heavily on abstinence-focused or biologically oriented teaching, which tends to exclude essential topics such as consent, healthy relationships, contraception, and the prevention of STIs (8). As a result, young people frequently enter adolescence with inadequate knowledge about reproductive health, contributing to higher rates of early sexual activity, unintended pregnancies, and STIs, including HIV (1, 6).

Nevertheless, research demonstrated that when culturally adapted CSE programs are implemented, they lead to improved knowledge, safer sexual behaviors, delayed initiation of sexual activity, and reduced health risks among adolescents (9). However, reproductive education in most developing countries face strong resistance from communities, and stigmatization surrounding discussions of sexuality led to lack of standardized curriculum (10). Thus, strengthening policy frameworks, increasing public awareness, and integrating evidence-based educational approaches remain critical steps toward improving adolescent SRH outcomes in developing countries.

In Kazakhstan, the introduction of sexuality education has been a topic of debate, shaped by sociocultural norms, political considerations, and varying levels of public acceptance. Although discussions on adolescent health have gained prominence due to rising concerns about early onset of sexual activity, elevated STI rates among youth, and limited reproductive health knowledge, the integration of formal sexuality education into the national school curriculum remains limited (11). Existing programs tend to be fragmented, inconsistently delivered, or focused narrowly on biological aspects without addressing essential psychosocial components such as consent, communication, and healthy relationships. As Kazakhstan seeks to modernize its public health and educational frameworks, it is essential to understand the effectiveness of sexuality education interventions globally and their relevance to the local context.

Therefore, the current study is a systematic review that aims to evaluate the impact of comprehensive sexuality education on adolescents' knowledge, attitudes, and sexual health behaviors, and to assess its applicability and potential integration within the Kazakhstani context. The review reports on evidence from both intervention studies (randomized and quasi-experimental) and observational studies examining associations between exposure to school-based sexuality education and sexual and reproductive health outcomes. Given the growing evidence that structured, age-appropriate sexuality education improves awareness, promotes safer sexual practices, and reduces risk-taking behaviors among young people, the study hypothesizes that adolescents exposed to CSE will demonstrate significantly higher levels of sexual health literacy and more protective behaviors compared to those receiving limited or no formal instruction. Furthermore, it is hypothesized that adapting such educational models to Kazakhstan's cultural, social, and educational environment will be both feasible and beneficial, supporting the development of evidence-based strategies to enhance adolescent sexual health in the country.

## Methods

The current systematic review was conducted to systematically assess the effectiveness and impact of CSE interventions in schools. Due to the limited number of randomized and quasi-experimental studies, the review also included observational studies examining associations between exposure to school-based sexuality education and sexual and reproductive health outcomes. Thus, both intervention effectiveness evidence and supportive observational findings were used in this review. The review process adhered to established methodological and reporting standards, specifically the Cochrane Handbook for Systematic Reviews of Interventions and the PRISMA guidelines, ensuring a rigorous and transparent approach (PROSPERO ID number: 1345698). Guided by these frameworks, the study formulated clear research questions, defined clear inclusion and exclusion criteria, and outlined standardized procedures for literature screening, data extraction, and database searching. In addition, we assessed the methodological quality of eligible studies, and assessed the potential risk of bias. Together, these steps provided a structured and evidence-based foundation for evaluating the impact of school-based CSE programs.

### Search strategy

Electronic databases, PubMed/PMC, Cochrane, Scopus, Web of Science, and Google Scholar were searched for implication of sexuality education on adolescents. Searches included material up to October 2025. Search terms combined subject headings and keywords for comprehensive sexual education; OR sex education; OR school-based sex education, and region/country terms: Kazakhstan, and/or Central Asia. The complete database-specific search strategies, including Boolean operators and keyword combinations used for each database, including (“sex education” OR “sexuality education” OR “comprehensive sexuality education” OR “school-based sex education”) AND (“adolescent” OR “youth” OR “teen” OR “student”) AND (“sexual health” OR “reproductive health” OR “STI” OR “contraception”. The full reproducible search strategies for all databases are provided in Supplementary Table 1.

#### Inclusion criteria

We included studies that meet the following criteria: (1) studies evaluating school-based or school-related sexuality education interventions or programs delivered to children or adolescents; (2) studies incorporating elements of CSE; and (3) studies reporting measurable outcomes related to sexual and reproductive health knowledge, attitudes, behaviors, or health outcomes. In addition to randomized and quasi-experimental intervention studies, observational studies that examined associations between exposure to school-based sexuality education and relevant outcomes were also considered eligible when they provided quantitative outcome data.

#### Exclusion criteria

included grey literature (e.g., conference posters and abstracts), studies lacking a clear focus on sex education, those providing insufficient data for analysis or missing key statistical results, as well as case reports and review articles. Note, due to the limited number of randomized intervention studies in this field, observational studies examining the association between exposure to school-based sexuality education and sexual and reproductive health outcomes were also included to provide broader contextual evidence.

### Data collection process

SD and GD independently carried out the literature search, study selection, and methodological quality assessment to ensure objectivity and reduce reviewer bias. After completing their evaluations, the reviewers compared their selections, and any discrepancies were resolved through discussion with AA and ZK. Study screening was conducted in two sequential phases: in the first phase, titles and abstracts were reviewed to identify potentially relevant studies; in the second phase, the full texts of the shortlisted articles were examined in details to confirm that they met all predefined eligibility criteria. This multistep process ensured a rigorous and transparent selection of studies for inclusion.

### Types of outcomes

The outcomes assessed in this review encompass multiple domains to capture the comprehensive impact of CSE interventions on adolescents. The primary outcomes include sexual and reproductive health knowledge, such as understanding of anatomy, contraception, STI prevention, and pregnancy risks; sexual behavior, including age at sexual activity, frequency and correctness of contraceptive use, and number of sexual partners; as well as biological outcomes, such as the incidence of STI and adolescent pregnancies. The secondary outcomes include attitude changes, including perceptions of gender equality, consent, and safe sexual practices, as well as psychosocial measures such as self-efficacy, communication skills, and attitudes toward risk-taking behaviors.

### Data extraction and risk of bias

The extracted data included the name of the first author; publication year; country; methodology; and key results. To evaluate the methodological quality of the included studies, a risk of bias analysis was performed. Briefly, for randomized controlled trials, the Cochrane Risk of Bias-2 tool (RoB-2), as described by Sterne et al. (12), was used. The tool assesses bias across five domains: (1) bias arising from the randomization process; (2) bias due to deviations from intended interventions; (3) bias due to missing outcome data; (4) bias in measurement of the outcome; and (5) bias in selection of the reported result (12). Each domain was rated as low risk, some concerns, or high risk following the decision algorithms provided in the RoB-2 guidance. Two reviewers independently evaluated each study, and discrepancies were resolved through discussion with a third reviewer. For non-randomized studies, the ROBINS-I (Risk of Bias in Non-Randomized Studies of Interventions) tool was used in accordance with the methodology described in Sterne et al. (13),. This tool evaluates seven domains of bias: confounding, selection of participants, classification of interventions, deviations from intended interventions, missing data, measurement of outcomes, and selection of reported results (13). Each domain was graded as low, moderate, serious, or critical risk of bias. Assessments were performed independently by two reviewers, and disagreements were resolved by consensus. Of note: a formal GRADE assessment was not conducted due to the heterogeneity in study designs, interventions, and outcome measures; instead, an overall qualitative assessment of evidence certainty was undertaken.

## Results

### Study selection and characteristics

**Figure 1:**
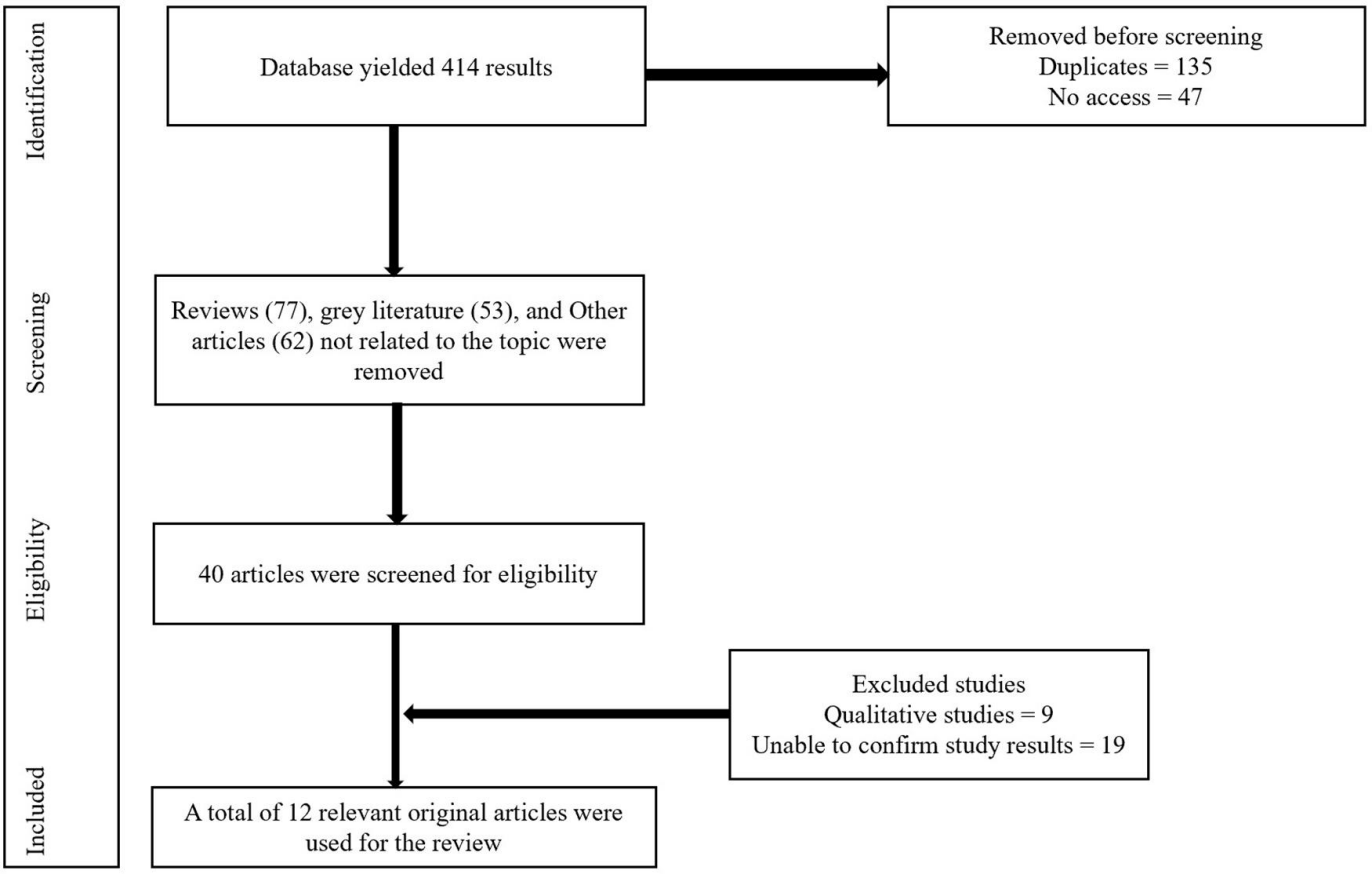
Flow diagram of the study selection process for the systematic review.

The systematic search identified a total of 414 records from electronic databases, which were screened according to the pre-defined eligibility criteria. After removing duplicates, and inaccessible articles, 232 full-text articles were evaluated for eligibility. Of these, twelve studies met the inclusion criteria and were included in the review Figure 1. The included studies comprised a combination of cluster-randomized controlled trials (*n* = 8), quasi-experimental intervention studies (*n* = 2), and observational designs including one cross-sectional and one longitudinal study (*n* = 2), reflecting a range of methodological approaches used to evaluate sexuality education programs. The studies were conducted across diverse geographical settings, including South Africa, the United Kingdom, the United States, China, Iran, Ethiopia, and Switzerland, and targeted adolescents typically aged 12-19 years Table 1. Sample sizes varied considerably, ranging from small pilot interventions of fewer than 100 participants to large cluster trials exceeding 1,000 students. Detailed characteristics of the included studies, including study design, sample size, setting, intervention type, and measured outcomes, are summarized in Table 1. The interventions evaluated included peer-led, teacher-led, and digitally delivered sexuality education programs, with program content addressing several outcome variables including HIV/STI prevention, contraception, reproductive health, sexual consent, and broader psychosocial skills Table 2. Most studies reported outcomes related to SRH knowledge, sexual attitudes, and behaviors, although the measurement tools and follow-up periods varied across studies. Follow-up assessments ranged from three months to several years, allowing evaluation of both short-term and sustained intervention effects. Accordingly, findings are presented as a narrative synthesis, and quantitative results are reported only as examples from individual studies to illustrate the range of observed effects to illustrate the direction and magnitude of effects rather than to imply quantitative synthesis. Hence, these estimates should not be interpreted as pooled, aggregated, or representative summary effects across studies.

**Table 1:** General characteristics of the selected studies. The table summarizes study design, sample size, geographic setting, participant age range, type of intervention, follow-up duration, and key outcomes. The included studies comprise of eight cluster-randomized controlled trials and four quasi-experimental or non-randomized studies, with sample sizes ranging from small pilot studies of less than 100 participants to large trials of more than 1,000 participants, which shows diverse intervention approaches across multiple countries.

No	Author(s)	Reference number	Country	Study Design	Sample Size	Age/Grade	Intervention	Comparator/Control	Key Outcomes
1	Jemmott JB 3rd et al., 2010	(14)	South Africa	Cluster RCT	1,057	12–14 yrs	School-based HIV/STD risk-reduction	Health-promotion control	Sexual behavior, condom use, HIV knowledge
2	Stephenson JM et al., 2004	(15)	England	Cluster RCT	∼8,000	13–14 yrs	Peer-led sex education	Teacher-led sex education	Sexual behavior, attitudes, knowledge
3	Stephenson JM et al., 2008	(21)	England	Cluster RCT	6,000+	13–14 yrs	Peer-led sex education	Teacher-led sex education	Pregnancy rates, abortion rates
4	Lohan M et al., 2023	(16)	UK	Cluster RCT	8,000+	13–15 yrs	Relationship & sexuality education engaging boys	Standard curriculum	Teenage pregnancy, contraceptive use, sexual behavior
5	Coyle K et al., 2021	(17)	USA	Group RCT	1,127	14–18 yrs	Comprehensive sexual health curriculum	Standard health education	Knowledge, attitudes, sexual behavior
6	Hu Z et al., 2023	(22)	China	Cluster RCT	1,312	10–12 yrs	School-based sexuality education	Usual curriculum	Knowledge, attitudes, sexual behavior
7	Hatami M et al., 2015	(19)	Iran	Quasi-experimental	120	14–16 yrs	Peer education on sexual health	No intervention	Knowledge, attitudes
8	Guo R et al., 2025	(18)	China	Cluster RCT	1,420	11–12 yrs	Online sexual & reproductive health program	Usual curriculum	Knowledge, attitudes, behavior
9	Constantine NA et al., 2015	(24)	USA	Cluster RCT	1,589	High school	Rights-based sexuality education	Standard health education	Knowledge, self-efficacy, intentions
10	Menna T et al., 2015	(20)	Ethiopia	Quasi-experimental	400	14–18 yrs	Peer education on HIV/AIDS	No intervention	Sexual behavior, HIV knowledge
11	Barrense-Dias Y et al., 2020	(25)	Switzerland	Cross-sectional survey	1,500	15–18 yrs	Various school sex education sources	Other sources (parents, peers)	Knowledge, attitudes, sexual behavior
12	Cavazos-Rehg PA et al., 2012	(23)	USA	Longitudinal analysis	N/A	14–18 yrs	State-level school sex education	No/limited sex education	Adolescent birth rates

**Table 2:** Summary of the characteristics of outcome variables. This table provides an overview of how sexual and reproductive health knowledge, attitudes, behaviors, and related outcomes were evaluated across different intervention designs and study settings.

No	Study	Outcome Variables	Type	Measurement/Assessment	Follow-up
1	(14)	HIV knowledge, condom use, sexual initiation, number of partners	Cognitive/Behavioral	Self-report questionnaire	12 months
2	(15)	Sexual knowledge, attitudes, sexual behavior (condom use, sexual initiation)	Cognitive/Attitudinal/Behavioral	Self-report, school surveys	1 year
3	(21)	Pregnancy rates, abortion, sexual behavior	Biological/Behavioral	Official records, self-report	6 years
4	(16)	Teenage pregnancy, contraceptive use, communication skills, sexual behavior	Biological/Behavioral/Cognitive	Self-report, school records	12 months
5	(17)	Knowledge, attitudes, sexual behavior, self-efficacy	Cognitive/Attitudinal/Behavioral	Standardized questionnaires	6 months
6	(22)	Sexual knowledge, attitudes, sexual behavior	Cognitive/Attitudinal/Behavioral	Structured questionnaires	6 months
7	(19)	Knowledge about STIs and HIV, attitudes toward sexual health	Cognitive/Attitudinal	Self-administered survey	3 months
8	(18)	Knowledge, attitudes, sexual behaviors (protective skills)	Cognitive/Attitudinal/Behavioral	Online pre- and post-tests	4 months
9	(24)	Knowledge, self-efficacy, behavioral intentions	Cognitive/Attitudinal	Structured survey	6 months
10	(20)	HIV knowledge, condom use, negotiation skills, sexual behavior	Cognitive/Behavioral	Self-report questionnaires	6 months
11	(25)	Knowledge, attitudes, sexual behavior	Cognitive/Attitudinal/Behavioral	Cross-sectional surveys	N/A
12	(23)	Teen birth rates	Biological	State-level administrative data	Multi-year

### Quality assessment: risk of bias

The methodological quality of the included studies was systematically assessed using the revised RoB-2 tool for randomized controlled trials and the ROBINS-I tool for non-randomized and quasi-experimental studies (12, 13). Among the eight randomized clinical trials, the majority were rated as low risk of bias in domains related to randomization and outcome measurement, though some studies demonstrated moderate risk due to incomplete outcome reporting or lack of blinding of participants and personnel. For example, the RIPPLE trials and FLASH curriculum studies reported robust randomization procedures and validated outcome measures, but self-reported behaviors introduced a potential for measurement bias Table 3. The risk of bias in the two quasi-experimental studies estimated to generally be moderate to serious, primarily due to non-randomized allocation and potential confounding variables that could influence outcomes. Peer-led interventions in Iran, Ethiopia, and China were limited by the absence of random assignment, and some studies had incomplete follow-up or lacked comprehensive reporting of statistical analyses. Overall, despite these limitations, most studies provided sufficient methodological detail to assess intervention effectiveness reliably. A summary of the risk-of-bias assessment across all included studies is presented in Table 3, which shows the distribution of assessment as low, moderate, and high-risk across each domain. These findings suggest that, while the included studies vary in methodological rigor, the overall quality is sufficient to support conclusions regarding the impact of school-based sexuality education on adolescents' knowledge, attitudes, and behaviors. In general, the certainty of evidence was considered moderate for knowledge and attitude outcomes, as these findings were supported by multiple randomized and quasi-experimental studies with generally consistent results. However, the certainty of evidence for behavioral outcomes was judged to be low to moderate, due to variability in outcome definitions, measurement approaches, and study designs.

**Table 3:** Risk of bias assessment of included school-based sexuality education studies. This table presents the overall risk-of-bias assessment for the included studies. Moderate outcome measurement bias was common, as most studies relied on self-reported sexual behaviors. Quasi-experimental and other observational designs generally showed higher risk due to confounding and lack of randomization, whereas cluster-randomized controlled trials typically demonstrated low to moderate overall risk when randomization procedures and allocation concealment were clearly reported. Cross-sectional and state-level observational studies were frequently rated as having a serious risk of bias related to confounding, given the number of external variables that could influence outcomes. Risk-of-bias ratings for each domain are defined as follows: Low risk, methods are robust and unlikely to bias results; Moderate risk, some concerns are present, but unlikely to substantially influence findings; Serious risk, notable methodological issues that may bias outcomes; Critical risk, major flaws likely to compromise the reliability of results; No information, insufficient detail to allow judgment.

No	Study	Study Design	Confounding/Randomization	Selection Bias	Classification of Intervention/Deviations	Missing Data	Outcome Measurement	Reporting Bias	Overall Risk
1	(14)	Cluster RCT	Low	Low	Low	Low	Moderate (self-report)	Low	Low-Moderate
2	(15)	Cluster RCT	Low	Low	Low	Moderate	Moderate (self-report)	Low	Moderate
3	(21)	Cluster RCT	Low	Low	Low	Moderate	Moderate (self-report)	Low	Moderate
4	(16)	Cluster RCT	Low	Low	Low	Low	Moderate	Low	Low-Moderate
5	(17)	Group RCT	Low	Low	Low	Low	Moderate	Low	Low-Moderate
6	(22)	Cluster RCT	Low	Low	Low	Low	Moderate	Low	Low-Moderate
7	(19)	Quasi-experimental	Moderate	Low	Low	Low	Moderate	Low	Moderate
8	(18)	Cluster RCT	Low	Low	Low	Low	Moderate	Low	Low-Moderate
9	(24)	Cluster RCT	Low	Low	Low	Low	Moderate	Low	Low-Moderate
10	(20)	Quasi-experimental	Moderate	Low	Low	Moderate	Moderate	Low	Moderate
11	(25)	Cross-sectional	Serious -confounding	Low	N/A	Low	Moderate	Low	Serious
12	(23)	Longitudinal/Observational	Serious -confounding	Low	N/A	Low	Low	Low	Serious

### SRH knowledge outcomes

A study by Jemmott et al. (14), reported that school-based CSE interventions demonstrated significant knowledge improvements of adolescents' SRH in South Africa. The study claimed that adolescents who participated in an HIV/STD risk-reduction program exhibited a mean knowledge score increase from 4.2 ± 1.1 to 7.6 ± 1.3 (on a 10-point scale) compared to minimal change in the control group (4.3 ± 1.2 to 4.9 ± 1.4, *p* < 0.001). The intervention group had significantly higher odds of correct responses regarding HIV/STI transmission, condom use, and abstinence knowledge, with an adjusted odds ratio (aOR) of 2.45 (95% CI: 1.62–3.71, *p* < 0.001) (14).

Analyzing data from 4,978 young adults, Barrense-Dias and colleagues reported that most participants relied on friends (38.9%) or parents (27.3%) for sex education, while fewer cited school (19.1%) or the internet (8.0%) as their main resource. Notably, individuals learned from school reported the lowest rate of sexually transmitted infections (6.8%), followed by those taught mainly by parents (8.2%), whereas reliance on internet sources (11.3%) or friends (11.7%) was associated with considerably higher STI rates (25). The cluster-randomized trial by Hu et al. (22), demonstrated that an internet-based sexuality-education intervention produced substantial improvements in students' sexual knowledge both the short-term *β* = 4.28 (95% CI: 4.05–4.52) and long-term *β* = 2.36 (95% CI: 2.13–2.60). The study also showed that students in the intervention group answered an average of 13 out of 20 sexual-knowledge questions correctly immediately after the program and 12 out of 20 one year later, compared to a baseline score of 8 correct answers (22). A similar finding reported by Constantine et al. (24), that showed students who received the CSE demonstrated significantly greater pre- to post-test improvements than the control group in sexual-health knowledge, with a standardized mean difference of 0.44 (24).

In the UK, Stephenson and colleagues examined the effectiveness of peer-led sex education in a school-based randomized trial of over 8,000 pupils across 29 schools in the RIPPLE peer-led programs, which showed notable improvements in sexual self-efficacy and attitudes toward contraception among participants compared to the control group (15). Interestingly, some of gains remained at a follow-up-study approximately 12 to 18 months later. Another study from the UK, the JACK trial by Lohan et al. (16),, reported improved attitudes toward delaying sexual initiation and contraceptive use (16). Similarly, Coyle and colleagues evaluated the effectiveness of a school-based comprehensive sexual health curriculum (FLASH) on sexual behavior and outcome of high-school students in the USA, and reported modest gains in condom-use self-efficacy and perceived social norms supporting safer sexual behavior, but greater gains among students who were younger or had no prior sexual experience (17).

Recently, Guo et al. (18), reported the results of a cluster randomized control trial using an online interventions in China, and reported that the mean knowledge scores increased from 56.3% ± 9.4% to 81.7% ± 8.7% post-intervention (*p* < 0.001), reflecting substantial improvements in understanding reproductive physiology, contraception, and safe sexual practices (18). In 2015, two separate peer-led interventions using validated sexual health questionnaires in Iran (19), and in Ethiopia (20), both reported significant increase in mean knowledge scores by approximately 15-18 points (*p* < 0.001). These findings indicate that CSE interventions, whether delivered through peer-led, teacher-led, or digital formats, effectively enhance adolescents' SRH knowledge across diverse cultural and geographic contexts. Knowledge gains were often maintained at follow-up, suggesting that these programs provide lasting benefits in adolescents' understanding of sexual health, contraception, and disease prevention.

### Sexual attitudes and psychosocial outcomes

CSE interventions demonstrated significant improvements in adolescents' sexual attitudes and psychosocial outcomes, with measurable effect sizes reported in several studies. Both of the RIPPLE studies in the UK, published in 2004 (15), and in 2008 (21), found that participants in peer-led programs, scored between 15%-20% higher on measures of sexual self-efficacy and attitudes toward contraception compared to control groups (*p* < 0.05), which were maintained in long-term follow-up, with knowledge retention rates of up to 18 months post-intervention. In the JACK trial, intervention participants reported a 22% increase in positive attitudes toward delaying sexual initiation and use of contraception relative to the control group, with an adjusted odds ratio of 1.45 (95% CI: 1.12-1.87, *p* = 0.004) for adopting safer sexual attitudes. This finding is aligned with the results of the FLASH program in the USA that produced a 13% improvement in condom use self-efficacy and a 17% increase in participants' perceived social norms regarding safe sexual behavior at 3-month follow-up (*p* < 0.05) (16).

Furthermore, the results of interventions from Iran and Ethiopia reported statistically significant gains in psychosocial outcomes, including self-efficacy, intentions to adopt protective behaviors, and attitudes toward HIV/STI prevention. For example, in Iran, Hatami et al. (19) reported a mean knowledge and attitude score increase from 62.4 ± 8.1 to 78.5 ± 7.3 among adolescent girls after peer-led interventions (*p* < 0.001) (19). In Ethiopia, Menna et al. (20) reported a 20% increase in positive attitudes toward condom use and delaying sexual activity post-intervention (20). The findings further, supports the theory that school-based sexuality education interventions effectively enhance adolescents' sexual attitudes and psychosocial readiness. The interventions fostered measurable shifts in self-efficacy, intention, and perception, which are critical precursors to adopting safer sexual behaviors.

### Sexual behaviors and health outcomes

In the South African study, participants in the HIV/STD risk-reduction program had significantly lower odds of engaging in intercourse (OR = 0.62, 95% CI: 0.42-0.94, *p* = 0.02), unprotected intercourse (OR = 0.51, 95% CI: 0.30-0.85, *p* = 0.01), and having multiple sexual partners (OR = 0.50, 95% CI: 0.28-0.89, *p* = 0.02) compared to the control group (14). The proportion of adolescents reporting sex at follow-up was 4.8% in the intervention group vs. 7.2% in the control group, while multiple sexual partners were reported by 1.8% vs. 3.2%, respectively (14).

The same findings reported in the RIPPLE peer-led intervention that reported reductions in self-reported sexual activity and risky sexual behaviors among adolescents. For instance, the percentage of sexually active students decreased by approximately 10%-12% relative to controls at twelve-month follow-up, and contraceptive use increased by 15% (15). These findings are mirrored in the JACK trial in the UK reporting a 19% reduction in teenage pregnancy rates in schools that implemented the intervention compared to control schools over a 24-month period (16). Cavazos Rehg PA and colleagues (23) examined the relationship between school-based sexuality education and adolescent birthrates using a state-level longitudinal analysis, and reported that school-based CSE is linked to reduced adolescent birthrates (*β* = −0.61; *P* = .001) (23).

Behavioral improvements such as, increased condom use, reduced number of sexual partners, and higher intentions to delay sexual initiation were reported amongst participants in interventions in most of the studies across different countries (22–25). For instance, in the FLASH in USA, authors reported an increase in condom use at last sexual intercourse from 62% to 77% among participants, and delayed initiation of sexual activity observed in 13% more students compared to controls (17). Collectively, these findings indicate that CSE interventions are effective in reducing risky sexual behaviors and improving reproductive health outcomes among adolescents.

## Discussion

The current review demonstrates that well-designed, CSE interventions are effective in improving adolescents' SRH knowledge, shaping safer attitudes, and reducing risky behaviors. These global evidence-based insights provide a strong foundation for considering how similar interventions might be adopted and adapted in the context of Kazakhstan, where sex education remains limited and contested. It does not currently form part of the national school curriculum (26). Although the subject of “valeology” (science of healthy living) covers some SRH topics, it lacks comprehensive, standardized content on contraception, consent, and gender equity. The public health code recognizes adolescents' right to reproductive education and protection, yet no formal policy mandates comprehensive CSE in schools (27).

While none of the included studies were conducted in Kazakhstan, several core components of CSE interventions, appear effective across diverse settings. These conclusions are drawn primarily from intervention studies, whereas observational studies included in the review provide complementary contextual evidence regarding associations between sexuality education exposure and sexual health outcomes. For example, structured curricula, skills-based learning, and integration within school systems, which may be considered broadly transferable. Evidence from successful interventions suggests that embedding CSE within existing subjects, combined with interactive and skills-oriented teachings, may enhance acceptability while maintaining educational effectiveness in conservative settings (28). However, other components, such as content related to sexual norms, delivery approaches (e.g., peer-led vs. teacher-led), and parental or community engagement, appear to be more context-sensitive and require cultural adaptation.

From an implementation-science perspective, the transferability of public health interventions across settings depends on the interaction between core intervention components and the local implementation context. Frameworks, such as the Consolidated Framework for Implementation Research and policy-transfer theory emphasize that while certain “core elements” of interventions, such as structured curricula, skills-based learning, and integration within school systems may remain stable across contexts, their successful implementation depends on adapting delivery mechanisms to local cultural norms, institutional capacity, and stakeholder acceptance (29). Applying this perspective to Kazakhstan suggests that evidence-based CSE programs could potentially be introduced by retaining core educational components, while modifying culturally sensitive aspects of program content, teacher training approaches, and community engagement strategies to align with local sociocultural expectations and policy environments.

In Kazakhstan, where sociocultural attitudes toward sexuality education may differ, careful adaptation of content and delivery mechanisms will be essential to ensure acceptability and effectiveness.

Despite the growing global evidence supporting the effectiveness of school-based sexuality education, the present review shows a lack in intervention research within Central Asia, including Kazakhstan. Most available studies in the region are qualitative, policy-focused, or situation analyses, examining the perceptions of parents, teachers, or program implementation barriers rather than measuring behavioral or biological outcomes. The absence of rigorous experimental or quasi-experimental studies evaluating the impact of comprehensive sexuality education in schools limits the ability to draw evidence-based conclusions about its effectiveness in this context. Therefore, there is an urgent need for well-designed, context-specific intervention research to assess the short and long term outcomes of CSE programs, that might improve adolescent sexual and reproductive health in the region.

Evidence from included studies suggests that CSE interventions are consistently associated with improvements in SRH outcomes, particularly in knowledge and attitudes, while behavioral outcomes show more variability across studies and contexts (14). Overall, effects tend to be more pronounced in cognitive domains compared to behavioral outcomes. This pattern suggests that while CSE may be more immediately effective in improving knowledge and attitudes, additional contextual and structural factors may be required to translate these gains into sustained behavioral change.

In comparison, available data indicate substantial gaps in SRH knowledge among adolescents in Kazakhstan. Nearly a quarter of young men reportedly lack any reproductive health information, while others rely on informal sources such as peers, family, or the internet (30). A national survey further showed low levels of HIV knowledge among adolescents, with only a small proportion answering all knowledge questions correctly, and many sexually active adolescents reporting symptoms of sexually transmitted infections without seeking medical care (31). These findings highlight important gaps that may be addressed through structured educational interventions.

However, implementing CSE in Kazakhstan will require navigating sociocultural sensitivities. For example, in a study that investigated teachers' attitudes towards sexual education in Karaganda region (North East of the country) reported that, while many support introducing sex education, they feel uncomfortable and unprepared to teach it (32). This perhaps reflects broader cultural resistance fueled by conservative traditions, moral framing of sexuality, and public unease about “discussing sex in schools” representing significant barriers, thereby efforts to introduce sexuality education have provoked public controversy in the past (33). Successful international models suggest that such barriers can be mitigated through phased implementation approaches, including teacher training, supportive guidance materials, and the gradual introduction of sensitive topics, as well as active engagement with parents and community stakeholders to improve social acceptability (34, 35).

Meanwhile, the government has committed to expanding youth-friendly health centres (YFHCs), and as of 2025, youth SRH services are being scaled up, with funding and training for providers to deliver nonjudgmental, confidential care (36). Also, the UNFPA-supported “Zanzu” safe space platform, which was launched in Kazakhstan and offers a multilingual, youth-friendly resource on puberty, contraception, and hygiene (37). These infrastructural developments can provide essential support for education-based strategies, ensuring that knowledge gained in classrooms can be reinforced through accessible services.

### Strengths and limitations

While the current review provides an analysis of evidence-based studies, there are a number of drawbacks. First, most of the studies reviewed come from considerably different sociocultural contexts, such Western Europe, sub-Saharan Africa, and their success factors may not fully translate. Second, there is a lack of published studies conducted within Kazakhstan or the Central Asia region, hence reducing the relevance and applicability of the findings to Kazakhstan, thus, future research in this area is warranted to address the issue. In addition, the applicability of these findings should be interpreted with caution, as certain intervention components may be transferable across settings, while others require careful cultural adaptation to align with local norms, educational structures, and policy contexts.

## Conclusion

In summary, the global evidence strongly supports the introduction of school-based CSE education as a means to boost knowledge, reshape attitudes, and reduce risky sexual behaviors among adolescents. In Kazakhstan, where formal sex education is sparse and SRH knowledge among youth is low, there is a compelling case for targeted, culturally adapted, pilot CSE programs, backed by teacher training, community engagement, and health service linkages. Doing so could address urgent public health needs, particularly adolescent pregnancy and STIs risk, and might lay the foundation for more comprehensive, sustainable SRH education in the country.

## Data Availability

The original contributions presented in the study are included in the article/Supplementary Material, further inquiries can be directed to the corresponding author.
