# Supplementary material for: The impact of implementation of sex education in schools, and its applicability in Kazakhstan
Source: Front Glob Womens Health. 2026 Apr 24;7:1761178. doi: 10.3389/fgwh.2026.1761178 (PMC13153124; doi:10.3389/fgwh.2026.1761178)
Supplement: Supplementary file 1 [file Datasheet1.pdf]

Supplementary Materials:

Table 1. General characteristics of the selected studies

No	Author(s)	Reference number	Country	Study Design	Sample Size	Age / Grade	Intervention	Comparator / Control	Key Outcomes
1	Jemmott JB 3rd et al. 2010	14	South Africa	Cluster RCT	1,057	12–14 yrs	School-based HIV/STD risk-reduction	Health-promotion control	Sexual behavior, condom use, HIV knowledge
2	Stephenson JM et al. 2004	15	England	Cluster RCT	~8,000	13–14 yrs	Peer-led sex education	Teacher-led sex education	Sexual behavior, attitudes, knowledge
3	Stephenson JM et al. 2008	21	England	Cluster RCT	6,000+	13–14 yrs	Peer-led sex education	Teacher-led sex education	Pregnancy rates, abortion rates
4	Lohan M et al. 2023	16	UK	Cluster RCT	8,000+	13–15 yrs	Relationship & sexuality education engaging boys	Standard curriculum	Teenage pregnancy, contraceptive use, sexual behavior
5	Coyle K et al. 2021	17	USA	Group RCT	1,127	14–18 yrs	Comprehensive sexual health curriculum	Standard health education	Knowledge, attitudes, sexual behavior

6	Hu Z et al. 2023	22	China	Cluster RCT	1,312	10–12 yrs	School-based sexuality education	Usual curriculum	Knowledge, attitudes, sexual behavior
7	Hatami M et al. 2015	19	Iran	Quasi-experimental	120	14–16 yrs	Peer education on sexual health	No intervention	Knowledge, attitudes
8	Guo R et al. 2025	18	China	Cluster RCT	1,420	11–12 yrs	Online sexual & reproductive health program	Usual curriculum	Knowledge, attitudes, behavior
9	Constantine NA et al. 2015	24	USA	Cluster RCT	1,589	High school	Rights-based sexuality education	Standard health education	Knowledge, self-efficacy, intentions
10	Menna T et al. 2015	20	Ethiopia	Quasi-experimental	400	14–18 yrs	Peer education on HIV/AIDS	No intervention	Sexual behavior, HIV knowledge
11	Barrense-Dias Y et al. 2020	25	Switzerland	Cross-sectional survey	1,500	15–18 yrs	Various school sex education sources	Other sources (parents, peers)	Knowledge, attitudes, sexual behavior
12	Cavazos-Rehg PA et al. 2012	23	USA	Longitudinal analysis	N/A	14–18 yrs	State-level school sex education	No/limited sex education	Adolescent birth rates

Table 2. Summary of the characteristics of outcome variables

No.	Study	Outcome Variables	Type	Measurement / Assessment	Follow-up
1	Jemmott JB 3rd et al., 2010	HIV knowledge, condom use, sexual initiation, number of partners	Cognitive / Behavioral	Self-report questionnaire	12 months
2	Stephenson JM et al., 2004	Sexual knowledge, attitudes, sexual behavior (condom use, sexual initiation)	Cognitive / Attitudinal / Behavioral	Self-report, school surveys	1 year
3	Stephenson JM et al., 2008	Pregnancy rates, abortion, sexual behavior	Biological / Behavioral	Official records, self-report	6 years
4	Lohan M et al., 2023	Teenage pregnancy, contraceptive use, communication skills, sexual behavior	Biological / Behavioral / Cognitive	Self-report, school records	12 months
5	Coyle K et al., 2021	Knowledge, attitudes, sexual behavior, self-efficacy	Cognitive / Attitudinal / Behavioral	Standardized questionnaires	6 months
6	Hu Z et al., 2023	Sexual knowledge, attitudes, sexual behavior	Cognitive / Attitudinal / Behavioral	Structured questionnaires	6 months
7	Hatami M et al., 2015	Knowledge about STIs and HIV, attitudes toward sexual health	Cognitive / Attitudinal	Self-administered survey	3 months

8	Guo R et al., 2025	Knowledge, attitudes, sexual behaviors (protective skills)	Cognitive / Attitudinal / Behavioral	Online pre- and post-tests	4 months
9	Constantine NA et al., 2015	Knowledge, self-efficacy, behavioral intentions	Cognitive / Attitudinal	Structured survey	6 months
10	Menna T et al., 2015	HIV knowledge, condom use, negotiation skills, sexual behavior	Cognitive / Behavioral	Self-report questionnaires	6 months
11	Barrense-Dias Y et al., 2020	Knowledge, attitudes, sexual behavior	Cognitive / Attitudinal / Behavioral	Cross-sectional surveys	N/A
12	Cavazos-Rehg PA et al., 2012	Teen birth rates	Biological	State-level administrative data	Multi-year

Table 3. Risk of bias assessment of included school-based sexuality education studies

No.	Study	Study Design	Confounding / Randomization	Selection Bias	Classification of Intervention / Deviations	Missing Data	Outcome Measurement	Reporting Bias	Overall Risk
1	Jemmott JB 3rd et al., 2010	Cluster RCT	Low	Low	Low	Low	Moderate (self-report)	Low	Low-Moderate
2	Stephenson JM et al., 2004	Cluster RCT	Low	Low	Low	Moderate	Moderate (self-report)	Low	Moderate
3	Stephenson JM et al., 2008	Cluster RCT	Low	Low	Low	Moderate	Moderate (self-report)	Low	Moderate
4	Lohan M et al., 2023	Cluster RCT	Low	Low	Low	Low	Moderate	Low	Low-Moderate
5	Coyle K et al., 2021	Group RCT	Low	Low	Low	Low	Moderate	Low	Low-Moderate
6	Hu Z et al., 2023	Cluster RCT	Low	Low	Low	Low	Moderate	Low	Low-Moderate
7	Hatami M et al., 2015	Quasi-experimental	Moderate	Low	Low	Low	Moderate	Low	Moderate
8	Guo R et al., 2025	Cluster RCT	Low	Low	Low	Low	Moderate	Low	Low-Moderate
9	Constantine NA et al., 2015	Cluster RCT	Low	Low	Low	Low	Moderate	Low	Low-Moderate

10	Menna T et al., 2015	Quasi-experimental	Moderate	Low	Low	Moderate	Moderate	Low	Moderate
11	Barrense-Dias Y et al., 2020	Cross-sectional	Serious - confounding	Low	N/A	Low	Moderate	Low	Serious
12	Cavazos-Rehg PA et al., 2012	Longitudinal / Observational	Serious - confounding	Low	N/A	Low	Low	Low	Serious

Supplementary table S1: Detailed searched strategies

**Database**	**Search String**
PubMed	("sex education" OR "sexuality education" OR "comprehensive sexuality education" OR "school-based sex education") AND (adolescent OR youth OR teenager OR student) AND (school OR curriculum OR program)
Scopus	TITLE-ABS-KEY ("sexuality education" OR "sex education" OR "CSE") AND TITLE-ABS-KEY (adolescent OR youth OR student)
Web of Science	TS= ("sexuality education" OR "sex education" OR "comprehensive sexuality education") AND TS=(adolescent OR youth OR student)
Cochrane	("sex education" OR "sexuality education") in Title/Abstract/Keywords

Google Scholar	"Comprehensive sexuality education" AND adolescent AND school
